# Guidelines for parenteral and enteral nutrition in geriatric patients in China

**DOI:** 10.1002/agm2.12110

**Published:** 2020-05-31

**Authors:** Mingwei Zhu, Hongyuan Cui, Wei Chen, Hua Jiang, Zijian Li, Birong Dong, Huaihong Chen, Yan Wang, Yun Tang, Yu Hu, Jianqin Sun, Yanjin Chen, Yexuan Tao, Suming Zhou, Weixin Cao, Junmin Wei

**Affiliations:** ^1^ Department of General Surgery Beijing Hospital Beijing China; ^2^ Department of Parenteral and Enteral Nutrition Beijing Union Hospital Beijing China; ^3^ Institute of Emergency and Disaster Medicine Sichuan Provincial People’s Hospital Chengdu China; ^4^ Department of Geriatrics Sichuan University West China Hospital Chengdu China; ^5^ Department of Neurology the Second Affiliated Hospital of Zhejiang University school of medicine Hangzhou China; ^6^ Department of Cardiovascular medicine Beijing Hospital Beijing China; ^7^ Department of General Surgery Chinese PLA General Hospital Beijing China; ^8^ Department of Geriatrics Zhongshan Hospital Fudan University Shanghai China; ^9^ Department of Nutrition Huadong Hospital Affiliated to Fudan University Shanghai China; ^10^ Department of General Surgery Tianjin Hospital of ITCWM Tianjin China; ^11^ Department of Clinical Nutrition Xinhua Hospital Affiliated to Shanghai Jiaotong University School of Medicine Shanghai China; ^12^ Department of Geriatrics Intensive Care Unit Nanjing Medical University First Affiliated Hospital Nanjing China; ^13^ Department of clinical Nutrition Shanghai Jiao Tong University Medical School Affiliated Ruijin Hospital Shanghai China

**Keywords:** elderly patients in China, enteral nutrition, parenteral nutrition

## Abstract

Based on the expert consensus on parenteral and enteral nutrition support for geriatric patients in China in 2013, domestic multidisciplinary experts were gathered to summarize the new evidence in the field of elderly nutritional support at home and abroad. The 2013 consensus was comprehensively updated and upgraded to a guideline by referring to the World Health Organization (WHO) guidelines for the Grading of Recommendations Assessment, Development, and Evaluation system for grading evidence. These guidelines were divided into two parts: general conditions and common diseases. After discussion by all members of the academic group and consultation with relevant experts, 60 recommendations were ultimately established as standardized nutritional support in the field of geriatrics in China.

## 1 INTRODUCTION

Based on the expert consensus on parenteral and enteral nutrition support for geriatric patients in China in 2013, Chinese scholars in the fields of evidence‐based medicine, public health, and clinical nutrition, and experts in related clinical disciplines were gathered to summarize the new evidence in the field of elderly nutritional support at home and abroad. The 2013 consensus was comprehensively updated and upgraded to guidelines by referring to the World Health Organization (WHO) Grading of Recommendations Assessment, Development, and Evaluation (GRADE) system for grading evidence.^1^, ^2^, ^3^ According to the GRADE system, the quality of evidence is categorized into four grades—“High quality: A,” “Moderate quality: B,” “Low quality: C,” and “Very low quality: D”—and the strength of the guidelines into two grades—“Strong” and “Weak.”

According to the requirements of the Delphi Method, the guidelines were sent to 100 Chinese experts in geriatrics, clinical nutrition, and nursing for comments, from which the guidelines were determined to have “mild agreement,” “moderate agreement,” or “strong agreement” according to the experts’ degree of agreement. Finally, the proportion of guidelines with “moderate agreement” plus those with “strong agreement” was taken as the percentage of agreement for the strength of the guidelines. The agreement results were listed next to each recommendation in the form of percentages to show the degree of support by the experts. All participants declared that they had not accepted the sponsorship or shares of any related corporation and that they did not hold any patents related to the field covered by each guideline.

## 2 NUTRITIONAL SUPPORT TEAM

Question: How do we establish a nutritional support team (NST) for elderly patients?

Recommendation 1: Multidisciplinary staff are required to establish an NST, led by geriatricians, other doctors, clinical nurses, nutritionists, and pharmacists, among others. (Evidence level B, strong recommendation, 97%)

Thus, geriatricians play an important role in organizing and running the multidisciplinary team. Nutritionists/dieticians, clinical pharmacists, physical therapists, and nurses are also key members. Surgeons, dentists, neurologists, psychologists, and other clinical specialists provide professional support. Several clinical trials have demonstrated that an NST can improve cost‐effectiveness and play an important role in decreasing the complications of nutritional support, costs, and length of hospital stay.^4^, ^5^ The main objectives of the NST are to provide reasonable nutritional support for elderly patients, including: (a) identifying malnutrition or nutrition risk; (b) formulating a reasonable nutritional support plan; (c) providing safe, reasonable, and effective nutritional support; and (d) monitoring and evaluating the effect of nutritional support.

## 3 DETERMINING ENERGY AND PROTEIN GOALS

Question: How do we determine the energy and protein requirements of elderly hospitalized patients?

Recommendation 2: The energy requirements of elderly hospitalized patients can be individually measured by indirect calorimetry (IC). (Evidence level C, weak recommendation, 90%)

Recommendation 3: If IC is not available or easily accessible, a simple weight‐based equation (eg, 20‐30 kcal/kg/d) can be used for most elderly patients. (Evidence level B, weak recommendation, 96%)

Recommendation 4: The protein intake of elderly hospitalized patients should be determined in accordance with the individual clinical situation. Daily protein intake can reach 1.0‐1.5 g/kg. Whey protein preparation is easier to digest. (Evidence level C, weak recommendation, 82%)

A variety of methods can be used to measure the energy requirements of elderly hospitalized patients. Resting energy expenditure (REE) is currently considered the gold standard for measuring human energy expenditure. In view of the significant individual differences in REE among the elderly, the energy expenditure should be measured in real time by IC, rather than by simply using formulas for estimation or prediction. In a study on energy expenditure estimation in elderly patients, it was found that the REE of hospitalized patients varied as a function of body mass index (BMI): the average REE of patients with BMI below 21 kg/m^2 ^was 21.4 kcal/kg/d, while the average REE of patients with BMI higher than 21 kg/m^2^ was 18.4 kcal/kg/d.^6^ Several guidelines at home and abroad have suggested that 20‐30 kcal/kg/d could be taken as the target amount for most elderly patients. Achieving the target energy intake can improve the long‐term prognosis of patients and reduce mortality.^1^, ^7^

For elderly patients, current dietary recommendations might underestimate their protein requirements. It is generally believed that elderly patients with normal renal function have a target protein intake of 1.2‐1.5 g/kg/d, and more significant clinical benefits could be obtained by increasing simple exercise activities. To seek the optimal protein intake level for the elderly, the multinational PROT‐AGE study, led by the European Union, has carried out extensive evidence‐based analysis and discussion. The study recommended at least 1.0‐1.2 g/kg of protein per day if safe and tolerable; for the elderly who perform regular exercise or activity, more protein is recommended (≥1.2 g/kg); the elderly with acute or chronic diseases need 1.2‐1.5 g/kg/d of protein intake. Patients with severe kidney disease but who are not on dialysis (glomerular filtration rate < 30 mL/min/1.73 m^2^) need to limit protein intake.^8^ In China, it is also recommended that the daily protein intake of elderly patients should be 1.0‐1.5 g/kg, and a certain amount of physical exercise and activity is recommended. As the absorption rate of whey protein is about twice that of casein, enteral nutrition (EN) formulations containing whey protein are more likely to meet the protein requirements of the elderly than EN formulations containing only casein.^1^

Question: How do we optimize the proportion of energy provided by fat and pharmacological supplements?

Recommendation 5: Elderly hospitalized patients who receive EN therapy should be given an appropriate preparation with the proportion of energy provided by fat based on disease status and gastrointestinal tolerance. (Evidence level A, strong recommendation, 99%)

Recommendation 6: The proportion of energy provided by fat can be increased appropriately in elderly patients receiving parenteral nutrition (PN) therapy (no more than 50% of non‐protein calories). (Evidence level C, weak recommendation, 99%)

Recommendation 7: Proper supplementation of glutamine can reduce infectious complications in elderly patients who receive nutrition therapy after surgery. We should also monitor the function of the liver and kidneys and limit the dosage of glutamine (≤0.5 g/kg/d). (Evidence level B, weak recommendation, 97%)

Recommendation 8: Omega‐3 fatty acid supplementation can be considered for elderly hospitalized patients within the scope of pharmacology, and it could improve the clinical prognosis. (Evidence level C, weak recommendation, 92%)

Current dietary guidelines for elderly Chinese residents state that the total fat intake of the elderly should account for 20%‐30% of their total energy consumption. In general, elderly patients should be given EN preparations with optimized fatty acid formulations, such as those containing higher chain fatty acids and omega‐3 fatty acids, which can help improve lipid metabolism. For some patients with partial intestinal malabsorption, severe exocrine pancreatic insufficiency, or severe hyperlipidemia, low‐fat EN preparations (energy from fat < 5%) can be considered. For critical patients and cancer patients, appropriately increasing the proportion of energy provided by fat is conducive to improving nutritional status.^1^ Elderly patients receiving PN treatment can receive increased fat for energy and reduced glucose, as appropriate, which can improve clinical outcomes. Breitkreutz’s study^9^ found that increasing the proportion of energy provided by fat in PN to 40%‐50% can meet the energy expenditure of patients without increasing risks, such as disturbances of blood glucose, respiratory failure, and changes in liver and kidney function. European parenteral nutrition guidelines for the elderly suggest that patients with hyperglycemia and cardiac and renal impairment might need to receive a higher fat content PN formula of up to 50% of total energy.^7^

For elderly patients with critical diseases and after major surgery, adding glutamine to the EN or PN formula can improve nutritional metabolism, maintain intestinal barrier function and immune function, and reduce serious complications, such as ectopic intestinal flora and infection.^10^, ^11^, ^12^ However, in the REDOx study,^13^ for patients with shock and multiple organ failure or hemodynamic instability requiring vasopressor support, excessively high doses of glutamine (>0.5 g/kg/d) can increase mortality. Given kidney function in the elderly declines with age, the tolerance for glutamine supplementation must be considered. A study has shown that oral supplementation with glutamine in elderly patients can lead to increased serum urea nitrogen and creatinine and a lower estimated glomerular filtration rate in elderly patients. Although it has no significant clinical significance, the renal function of patients should be monitored and the dose be limited to below 0.5 g/kg/d.^14^

Many studies and guidelines at home and abroad have suggested that reducing some omega‐6 fat emulsions and increasing pharmacological doses of omega‐3 fatty acids (such as fish oil fat emulsions) in PN formulas can decrease the level of inflammatory factors, reduce the incidence of infection and systemic inflammatory response syndrome, and shorten the length of hospital stay.^7^, ^15^ A systematic review on the use of fish oil for 6‐52 weeks and an oral dose of 0.03‐1.86 g/d concluded that oral fish oil supplementation for the elderly has a lower risk of adverse reactions and serious adverse reactions and is clinically safe within the dose range of this study.^16^

## 4 NUTRITIONAL SCREENING AND ASSESSMENT

Question: How do elderly patients receive nutritional screening and assessment?

Recommendation 9: The incidence of malnutrition in elderly patients is high and regular nutritional screening is recommended. The mini nutritional assessment‐short form (MNA‐SF) and nutritional risk screening (NRS2002) nutritional screening tools are useful. (Evidence level A, strong recommendation, 97%)

Recommendation 10: The nutritional status of elderly patients should be comprehensively assessed, including disease severity, eating conditions, laboratory tests, weight and body composition measurements, and an overall health assessment. (Evidence level C, strong recommendation, 97%)

In multiple guidelines at home and abroad, the NRS2002 is recommended as a useful screening tool for nutritional risk among inpatients, including the elderly. The MNA‐SF is based on six simple questions regarding medical history, weight, diet assessment, and a simple physical examination, which together determine whether a patient has malnutrition or is at risk of malnutrition. The assessment should be carried out as early as possible to achieve better clinical outcomes.^1^, ^7^ Zhu et al used the NRS2002 and MNA‐SF methods to conduct a nutritional screening survey for inpatients in 34 large hospitals in 18 major cities in China.^17^ It was found that 51.41% of elderly inpatients had nutritional risks, and that complications and lengths of stay of elderly patients with nutritional risks were higher than those not at nutritional risk.^17^ Skipper et al published an evidence‐based analysis of the sensitivity and specificity of the MNA‐SF, NRS2002, subjective global assessment (SGA), and other tools and found that the MNA‐SF was more conducive to the assessment of elderly patients than the other tools.^18^

A comprehensive nutritional assessment explains and expands on information obtained from nutrition screening. Nutrition professionals analyze and evaluate clinical information, and comprehensively judge medical and nutritional intake history, digestion, and absorption capacity, data from the physical examination, anthropometric and body composition analysis, biochemistry indicators, clinical manifestations, and other nutrition‐related problems to obtain a disease‐related nutritional diagnosis. Zhang et al^19^ systematically evaluated studies on the application of blood biochemical indicators to the diagnosis of malnutrition in elderly patients, including 111 studies and 52 911 patients, and found that serum albumin, cholesterol, hemoglobin, and prealbumin measurements were directly related to a diagnosis of malnutrition, and that these measurements were reduced in the malnutrition group. In 2016, the American Society for Parenteral and Enteral Nutrition (ASPEN) and the European Society for Clinical Nutrition and Metabolism (ESPEN) formed a preliminary consensus on how to diagnose malnutrition. The conclusion was that the diagnosis of malnutrition should be based on nutrition screening and that recent weight loss was the most important predictive factor.^20^ In 2018, the Global Leadership Initiative on Malnutrition jointly developed by ASPEN and ESPEN achieved a high degree of consistency in identifying malnutrition in elderly patients.^21^ Malnutrition is an important indicator of the comprehensive problems of elderly patients. In addition to the evaluation of malnutrition‐related indicators, a comprehensive assessment should also include physical functional status, mental and psychological status, an assessment of weakness and sarcopenia, pain, comorbidities, medications, social support, sleep disorders, vision, hearing, oral cavity, taste, and other factors that impact nutrition in the elderly.

## 5 ENTERAL NUTRITION TREATMENT

Question: What are the goals and indications for EN in elderly patients?

Recommendation 11: EN should be the first choice for elderly patients with malnutrition or nutritional risk and with normal or almost normal gastrointestinal function. A reasonable EN plan should be developed according to the patient’s individual characteristics to improve nutritional status, maintain organ function, and improve clinical outcomes. (Evidence level A, strong recommendation, 97%)

Before nutritional support is implemented, hypovolemia, low sodium, low potassium, and other electrolyte and acid‐base balance disorders must be corrected. Nutrition formulas, the optimal route for EN, and appropriate energy and nutrient levels should be tailored to each patient by taking into account age, nutritional risk, fasting, primary diseases, and different courses of the same disease as well as heart, lung, and kidney function, and other comorbidities. During EN, monitoring is important to assess its effectiveness and organ function and whether the nutritional plan needs adjustment. EN is the first choice for elderly patients with normal gastrointestinal function. PN is an alternative route in cases of gastrointestinal function deficiency and intolerance of the glycemic index tract.^1^, ^7^, ^22^, ^23^

Question: How do we choose EN formulas for elderly patients?

Recommendation 12: Standard total protein EN formulas are suitable for most elderly patients. Long‐term use of optimized fatty acid EN formulas can reduce the risk of cardiovascular events. (Evidence level B, strong recommendation, 97%)

Recommendation 13: Dietary fiber added to formula can reduce the incidence of diarrhea and constipation in patients with tube‐feeding EN, and dietary fiber intake ≥25 g/d can reduce constipation and improve clinical outcomes in patients with tube‐feeding. (Evidence level A, strong recommendation, 88%)

Standard total protein formulas are suitable for most elderly patients, and the amino acid or short peptide EN formulas are best for patients with gastrointestinal function deficiency (such as severe acute pancreatitis). Formulas with high energy density can improve the nutritional status of elderly patients. Whey protein can promote protein synthesis and weaken protein synthesis resistance in older people, and it provides more essential amino acids than casein. Older patients are likely to suffer from lactose intolerance due to lactase deficiency, and diarrhea is common. Formulas without lactose can be used for these patients. Reducing the use of formulas rich in saturated fatty acids and increasing the amount of medium‐chain fatty acids and monounsaturated fatty acids is recommended for the elderly, as all of these supply energy rapidly and reduce the metabolic burden to the liver. Such formulas can also reduce the risk of cardiovascular disease in long‐term management.^24^ Dietary fiber can improve the intestinal function of elderly patients who receive tube‐feeding EN for extended periods of time, and fiber can also reduce the incidence of diarrhea and constipation. The Chinese Dietary Guidelines recommend a daily intake of dietary fiber of 25 g/d. A recent meta‐analysis showed that long‐term sufficient dietary fiber intake improved clinical outcomes.^25^

Question: How should elderly patients use oral nutritional supplements (ONSs)?

Recommendation 14: For elderly patients with malnutrition or at risk of malnutrition, supplementing ONSs with diet can improve nutritional status without affecting normal food intake. (Evidence level A, strong recommendation, 95%)

Recommendation 15: ONSs 400‐600 Kcal and/or 30 g protein per day and oral administration between meals for 30‐90 days can improve the nutritional status and clinical outcomes of elderly patients. (Evidence level A, strong recommendation, 96%)

Recommendation 16: A higher protein ONS formula might reduce the risk of complications and pressure sores in elderly hospitalized patients and can improve muscle strength and quality of life of elderly patients with sarcopenia. For elderly patients with hip fractures and orthopedic surgery, perioperative ONSs can reduce postoperative complications. (Evidence level A, strong recommendation, 92%)

Recommendation 17: High‐protein ONS formulas with beta‐hydroxy‐beta‐methylbutyrate complex (HMB) can contribute to increased muscle mass and improved quality of life for elderly hospitalized patients. (Evidence level B, weak recommendation, 82%)

Recommendation 18: By adjusting the taste of formulas, providing psychological counseling, and combining multiple strategies for encouragement, the compliance of elderly patients with ONSs can be improved. (Evidence level C, strong recommendation, 92%)

ONSs should be the preferred nutritional intervention for elderly patients with malnutrition or at risk of malnutrition, and when the regular diet cannot meet the requirements of the total body amounts (<60% of the target amounts). ONSs have the advantages of simplicity, convenience, and low price, and can satisfy the psychological desire of elderly patients to consume nutrition orally. In most cases, total nutrition formulas are recommended when using ONSs, including EN formulas or food for special medical purposes. ONSs can be used either as a substitute for some foods in the diet or as a supplement to increase intake and provide 400‐600 kcal daily, and oral administration between meals is considered the standard nutritional intervention therapy for ONS.^26^ Huynh et al^27^ gave ONSs (432 kcal/d) for 12 weeks to 106 elderly patients who were at nutritional risk. Compared with a control group, they gained weight and improved their BMI (*P* = 0.0009). In two Chinese studies,^28^, ^29^ ONSs (500 kcal/d, oral administration between meals) were given to patients receiving neoadjuvant chemotherapy for 90 days after gastrointestinal cancer surgery, and bodyweight and BMI improved. Philipson et al^30^ conducted a large‐sample retrospective study, including 197 677 hospitalized patients. The ONS group had an average 2.3 days shorter duration of hospital stay (95% confidence interval, −2.4 days to −2.2 days) or a 21.0% shorter stay, and had $4734 less in medical costs (95% confidence interval, −$4754 to −$4714) or 21.6% lower cost. Stratton et al^31^ performed a meta‐analysis from eight randomized control trials (RCTs) on ONSs in elderly patients, and the ONS group had a significantly reduced 6‐month readmission rate among elderly patients.

In a systematic review of 36 RCTs, a high‐protein ONS formula had clinical, nutritional, and functional benefits, including reduced complications and readmission rates, improved grip strength, and increased intake of total energy and protein.^32^ A higher‐protein ONS formula also significantly improved muscle strength and quality of life in elderly patients with sarcopenia.^33^ In an RCT study including 652 elderly hospitalized patients with SGA scores of B and C, the 90‐day mortality rates in the intervention group with high HMB supplementation were significantly lower than those of the control group, and the intervention group achieved better nutritional status (according to the SGA classification) on the 90th day, as well as significant weight gain on the 30th day.^34^ Recent studies have found that elderly patients undergoing ONS obtained benefits including a significant improvement in their quality of life and a reduction in complications and costs.^35^

A long‐term study of compliance with ONS in cancer patients by Bolton et al^36^ found that 54% of patients stopped due to disliking the taste. Related factors influencing the overall evaluation of ONSs were as follows: taste, aroma, appearance, taste after drinking, flavor intensity, sweetness, and thickness. Although more than 10 flavors of ONS formulas, such as vegetables, fruits, chocolates, strawberries, and coffee, have been developed abroad, there is still a big difference in taste compared to natural diets.

Question: How do we choose the route of enteral tube‐feeding?

Recommendation 19: Nasogastric tubes are suitable for elderly patients who receive short‐term tube‐feeding (2‐3 weeks). Elevating the head to 30‐45 degrees can prevent aspiration pneumonia. (Evidence level C, strong recommendation, 99%)

Recommendation 20: For elderly patients undergoing major abdominal surgery who are expected to need long‐term postoperative tube‐feeding, placing a jejunostomy, or nasogastric tube during the operation is recommended. When a proximal gastrointestinal anastomosis is performed, EN can be performed through a jejunal nutrition tube placed at the distal end of the anastomosis. (Evidence level C, weak recommendation, 92%)

Recommendation 21: For elderly patients who need long‐term nutritional support, percutaneous endoscopic gastrostomy (PEG) is recommended over a nasogastric tube. PEG is recommended for tube‐feeding EN that is expected to be used for more than 4 weeks. (Evidence level A, strong recommendation, 97%)

Recommendation 22: For patients at high risk of aspiration pneumonia, jejunal tube placement should be performed by a variety of routes (such as nasojejunal tube, jejunostomy, or percutaneous endoscopic jejunostomy). (Evidence level C, weak recommendation, 92%)

Tube‐feeding can meet energy and nutrient requirements and improve nutritional status for elderly patients.^1^, ^7^ The selection principles for the various routes include the following: the choice should suit EN; the insertion procedure should be easy and convenient with minimal injury to patients; and the choice should be comfortable and conducive to long‐term tube‐feeding if that is required. Nasogastric tubes are the most commonly used EN route. Nasogastric tubes are suitable for elderly patients who receive short‐term tube‐feeding (2‐3 weeks). Elevating the head to 30‐45 degrees can prevent aspiration pneumonia.^1^ For patients receiving proximal gastrointestinal anastomosis, the placement of a jejunal feeding tube at the distal end of the anastomosis can reduce the impact on the gastrointestinal anastomosis and is conducive to implementing early EN.^1^ Studies have confirmed that PEG is superior to nasogastric tubes because it provides more energy, is better at maintaining or improving nutritional status, and is not involved in tube displacements or reinsertions. A few studies have found improvements in the quality of life of patients with PEG and no difference in mortality, but the incidence of aspiration pneumonia in patients undergoing PEG is lower than that with nasogastric tubes. Therefore, if EN is anticipated for longer than 4 weeks, and there are no contraindications and the consent of the patient or family members is obtained, PEG is recommended. Skilled endoscopic ability can reduce the complications of PEG.^1^, ^7^

## 6 PARENTERAL NUTRITION TREATMENT

Question: Which elderly patients need to receive PN?

Recommendation 23: Total parenteral nutrition (TPN) is recommended for elderly patients with severe gastrointestinal dysfunction or an inability to use EN. (Evidence level B, strong recommendation, 97%)

Recommendation 24: When EN cannot provide 60% of the total energy and protein intake needed by the patient, supplementary parenteral nutrition (SPN) should be administered to meet the energy and protein requirements of elderly patients, maintain nutritional status and organ function, and improve clinical outcomes. (Evidence level A, strong recommendation, 99%)

Severe gastrointestinal dysfunction is common in diseases such as severe abdominal infection, severe acute pancreatitis, intestinal obstruction, severe inflammatory bowel disease, high intestinal fistula, short bowel syndrome, and intestinal ischemia. The gastrointestinal tract has the basic functions of digestion and absorption but cannot be used in cases of refractory vomiting or diarrhea, or serious mental or psychological disorders. TPN is the only means for elderly patients with the above conditions to obtain nutrients and sustain life. SPN refers to a mixed nutritional support treatment method in which part of the energy and protein requirements are supplemented by PN when EN is insufficient. The advantage of SPN is that while EN maintains the intestinal barrier function, supplementing PN can meet the needs for energy and protein. This approach can promote protein synthesis, quickly correct undernutrition, or maintain nutritional status to achieve the goal of improving clinical outcomes. A multicenter survey conducted in 26 intensive care units (ICUs) in China found that if only EN was given, only 31.8% of surgical patients achieved the target feeding amounts.^37^ Heyland et al^38^ surveyed 3390 ICU patients in 201 centers and found that 74.0% of patients failed to reach 80% of the target energy intake, and protein supply was only 57.6% of the target amount. The RCT study conducted by Heidegger et al^39^ showed that for critically ill patients whose EN did reach the target feeding amount of 60%, SPN was given on the 4th‐8th days after entering the ICU, and nearly 100% of the energy supply reached the targets. Compared with continuous EN, the 28‐day nosocomial infection rate of the SPN group was significantly reduced (*P* = 0.0338). In recent years, additional studies have reached similar conclusions, which could be attributed to the fact that when the energy supply of EN is less than 60% of the target amount, it directly affects the nutritional status and organ function of elderly patients and results in increased complications.^40^ In this case, the advantages of SPN in improving energy and protein supply are highlighted. Protein anabolism is promoted, thus maintaining the functions of tissues, cells, and organs, and promoting the repair of autophagy in severe conditions.

Question: When do elderly patients start using PN?

Recommendation 25: For elderly patients with a normal nutritional status at admission, and for whom EN cannot meet more than 60% of nutritional requirements for more than 7 days, it is recommended to start PN. For elderly patients with moderate or higher malnutrition who cannot eat normally or obtain sufficient nutrients through EN within 72 hours after admission, it is recommended to start PN. (Evidence level B, strong recommendation, 97%)

Recommendation 26: For elderly critically ill patients with low nutrition risk (NRS2002 ≤ 3 points or Nutrition Risk in Critically Ill [NUTRIC] score ≤ 5 points), start PN when EN fails to reach the 60% target feeding amount on the 7th day after surgery. For those with high nutrition risk (NRS2002 ≥ 5 points or NUTRIC score ≥ 6 points), it is recommended to start PN when EN does not reach the target amount within 72 hours after entering the ICU. (Evidence level A, strong recommendation, 97%)

Elderly patients with a good nutritional status can usually tolerate insufficient intake for up to 7 days. In view of the many complications of PN, premature administration of PN might not be worth the cost. The results of the Early versus Late Parenteral Nutrition in the Pediatric Intensive Care Unit study^41^ showed that SPN started at 8 days after surgery reduced complications and hospital stay compared with starting it 48 hours after the operation. However, 60% of these operations were cardiac surgery, and patients with a BMI < 20 were excluded, thus, the conclusion is controversial. Two subsequent studies suggested that early implementation of SPN can reduce complications, such as infection, in critically ill patients with an EN supply that meets less than 60% of the target amount.^39^, ^42^ The conflicting results are related to the type of disease, disease severity, and preoperative nutritional status. Jie et al^43^ found that patients at low nutritional risk (NRS2002 score 3‐4 points) and who were given nutritional support did not receive a clear benefit, while those at high nutritional risk (NRS2002 score ≥ 5) had significantly reduced infectious and non‐infectious complications. The study conducted by Heyland et al^44^ showed that the nutritional support effect for critical patients with NUTRIC scores ≥ 6 was significantly better than that for patients with a NUTRIC score ≥ 5. Therefore, the optimal starting time of SPN varies depending on the severity of the disease and the patient’s nutritional status, and the goal of improving the outcome can best be achieved by standardized application.

Question: Are there special requirements for PN prescription in elderly patients?

Recommendation 27: PN in elderly patients should be premixed with various nutrients and then infused in “all‐in‐one” solutions to reduce the incidence of metabolic complications. (Evidence level B, strong recommendation, 99%)

Recommendation 28: The compounded PN prescription is in line with the principle of individualized treatment and is suitable for elderly patients with special needs. Industrialized multichamber bags can reduce bloodstream infections and are suitable for long‐ and short‐term application of PN to elderly patients. (Evidence level A, strong recommendation, 97%)

The PN prescription for elderly patients should be determined according to the metabolic characteristics of the patient and the total energy and protein supply should include the intake of EN. The nutrients should be selected according to those with the least impact on the functions of the liver and kidneys as much as possible, and with enough essential nutrients to meet the metabolic needs. “All‐in‐one” is a method of infusion after mixing all the nutrients required by the patient and has the advantages of complying with physiology, promoting body protein synthesis, reducing the concentration and osmotic pressure of individual nutrients, reducing the metabolic load on organs, such as the liver and kidneys, and reducing metabolic complications. Pan et al^45^ confirmed that the “all‐in‐one” model reduced the incidence of treatment‐related adverse events by 44% compared to single‐bottle infusions. Industrialized multichamber bags have the advantages of reducing prescription and configuration errors, fewer impurities, less microbial contamination, convenience of use, and requiring fewer health‐care professionals. A cohort study including more than 70 000 patients^46^ showed that multichamber bags significantly reduced the incidence of bloodstream infections compared to a premixed PN group (*P* < 0.01). In another prospective, randomized controlled multicenter Evaluating the Influence of Ready‐to‐use Parenteral Nutrition In the Clinical Outcome of Patients study,^47^ the number of blood infections and central‐catheter‐related blood infections in the multichamber bags group was significantly lower than that in the preparation group (*P* = 0.03 and *P* < 0.0001, respectively). Thus, the multichamber bag, containing a standardized PN solution, can improve safety and clinical convenience and maximize the effect of nutritional treatment on surgical patients with stable conditions who are receiving short‐term PN treatment.

Question: Are vitamins and microelements routinely used in elderly patients’ PN prescriptions?

Recommendation 29: Vitamins are the basis for the effective use of glucose and fatty acids for energy and protein synthesis. PN prescriptions for elderly patients should include regular doses of intravenous, fat‐soluble, and water‐soluble vitamins. (Evidence level B, strong recommendation, 99%)

Recommendation 30: In the PN support program for elderly patients, intravenous multiple trace elements preparations should be routinely added. (Evidence level C, weak recommendation, 86%)

A study has found that the intravenous supplementation of multivitamins after abdominal surgery increased the total antioxidant capacity of the patients, reduced systemic inflammation, and promoted wound healing compared to the control group.^48^ A meta‐analysis including 21 RCTs showed that antioxidant micronutrients (antioxidant vitamins and trace elements) significantly reduced total mortality and the incidence of infectious complications in critically ill patients, with a trend toward a reduced time of mechanical ventilation, with no influence on ICU time or length of stay.^49^ To prevent Wernicke encephalopathy and refeeding syndrome, elderly patients receiving PN should be supplemented with multivitamins in regular doses, while critically ill patients should be supplemented with double doses.^50^ According to the latest Chinese guidelines,^51^ the supplementary amount of manganese, copper, and chromium in trace elements preparations should be 55 μg/d, 0.3‐0.5 mg/d, and 0.14‐0.87 μg/d, respectively.

Question: How do we choose the route of PN infusion in elderly patients?

Recommendation 31: Peripheral intravenous infusion is the first choice for short‐term SPN in elderly patients. The osmotic pressure of PN nutrient solutions should not exceed 900 milliosmols per liter (mOsm/L), but the occurrence of superficial phlebitis should be prevented as much as possible. (Evidence level C, weak recommendation, 84%)

Recommendation 32: High osmotic pressure (>900 mOsm/L) or long‐term PN (>14 days) is recommended to be administered via central venous infusion. Percutaneous central venous catheterization is suitable for critically ill patients and the subclavian vein route is preferred, but it is not recommended for longer than 30 days. Peripherally inserted central catheter (PICC) has a low risk of puncture complications and fewer infectious complications and should be the main route for PN infusion in elderly patients. (Evidence level B, weak recommendation, 83%)

Multiple clinical trials and studies have confirmed that peripheral parenteral nutrition (PPN) is safe and effective for hospitalized patients, especially perioperative patients.^52^ Indications for PPN include short‐term PN treatment, SPN with low energy and nitrogen, or an inability to perform central venous PN. It is generally believed that the final osmotic pressure of parenteral nutrition solution via PPN should not exceed 900 mOsm/L, while the amino acid concentration should not exceed 3% and the glucose concentration should not exceed 10%.^53^ Several guidelines suggest that the nutrient solution osmotic pressure can reach 850‐900 mOsm/L via the PPN route.^1^, ^7^, ^54^

A cohort study of 3471 patients demonstrated a significant reduction in duct‐related infections and thrombotic complications in the subclavian vein route, despite higher mechanical complications compared with the internal jugular vein and femoral vein routes (*P* = 0.047 and *P* = 0.02, respectively).^55^ Compared with central venous catheterization, PICC has fewer complications and a higher success rate. With the widespread application of ultrasonic technology in deep venipuncture in recent years, the puncture site has been changed from the inferior vessel of the elbow to the brachial vein of the elbow in PICC catheterization, which has significantly reduced the occurrence of mechanical phlebitis.^52^

## 7 MONITORING AND MANAGEMENT OF PARENTERAL AND ENTERAL NUTRITION

Question: Is monitoring needed for EN in elderly patients?

Recommendation 33: Prior to EN in patients who are at risk of refeeding syndrome, a routine examination of electrolytes and metabolic products should be performed and any disorders of water electrolyte or vitamin B1 should be corrected. The metabolic indexes should be monitored during EN. (Evidence level B, strong recommendation, 88%)

Recommendation 34: EN through the nasogastric tube should be regularly monitored for residual gastric volume. If the gastric residual volume is significant (>250 mL), consider adjusting the EN method by changing the placement of the tube, reducing the frequency of feeding, switching to feeding routes, or stopping EN. (Evidence level C, strong recommendation, 96%)

Elderly patients at risk of refeeding syndrome can have abnormal serum levels of potassium, phosphorus, magnesium, vitamin B1, and retention of water and sodium, all of which should be corrected before EN.^56^ Nutritional support should be staged, that is, 25% of the total amount given at the beginning and the balance achieved 3‐5 days later. Changes in water and electrolytes should be closely monitored. High‐risk factors for aspiration pneumonia due to gastroesophageal reflux include disturbances of consciousness, inadequate posture, sedation, critical illness, vomiting, and gastric retention. Monitoring of gastric residual volume is also related to the prevention of aspiration pneumonia. Some studies have found that when the gastric residual volume is greater than 250 mL and the patient has more than one risk factor, or when the residual volume is more than 200 mL and the patient has more than two risk factors, adjusting the EN method should be considered. These adjustments could include a change in intubation position, a decrease in infusion speed, a change to PEG/percutaneous endoscopic jejunostomy, or an end to EN.^57^

Question: Is it necessary to routinely monitor complications during the implementation of PN in elderly patients?

Recommendation 35: During the implementation of PN in elderly patients, we should routinely monitor liver and kidney function, blood lipids and glucose, and other metabolic characteristics, especially for those at high risk of refeeding syndrome. Standard preventive measures can reduce the incidence of complications. (Evidence level B, strong recommendation, 96%)

Recommendation 36: Complications such as bloodstream infections and catheter‐related infections are key monitoring issues in the implementation of PN in elderly patients. A catheter terminal culture is recommended when catheter‐related bloodstream infection is suspected. For this, venous blood is extracted and cultured through percutaneous and catheter channels. Prophylactic use of antibiotics is not beneficial in preventing catheter‐related infections. (Evidence level B, strong recommendation, 95%)

Recommendation 37: Elderly patients with long‐term TPN treatment are prone to PN‐related liver disease. Early resumption of food intake or EN and control of infections are important preventive methods. (Evidence level C, strong recommendation, 86%)

The incidence of PN complications in elderly patients is higher than that in young people. A carbohydrate infusion rate over 4‐5 mg/kg/min can lead to hyperglycemia, fat accumulation, and liver fat infiltration. When PN is applied, the glycemic control target is 6.1‐8.3 mmol/L.^1^, ^7^ Siegman et al^58^ suggested that it was only necessary to do a catheter culture when a bloodstream infection was suspected, and venous blood should be extracted through the percutaneous route and catheter at the same time for conventional bacterial culture. Prophylactic antibiotics, either locally or systemically, have no advantage in preventing catheter‐related infections. The risk factors for PN‐related liver disease include PN formula, malnutrition, deficiency of essential fatty acids, excessive energy intake, imbalance of amino acid intake, excessive fat intake, choline deficiency, overgrowth of intestinal bacteria, endotoxemia, deficiency of carnitine, and lack of EN.^59^

## 8 NUTRITIONAL SUPPORT TREATMENT OF COMMON DISEASES AMONG THE ELDERLY

### 8.1 Cardiovascular disease in the elderly

Question: How do we provide nutritional support for elderly patients with heart failure?

Recommendation 38: Nutritional counseling interventions can improve the clinical prognosis of elderly patients with chronic heart failure. EN is the first choice for nutritional support therapy. If there is severe gastrointestinal dysfunction, PN can be employed instead. Excessive fluid should be avoided and high‐energy‐density EN formulas are helpful for fluid management. (Evidence level B, strong recommendation, 92%)

A meta‐analysis of RCT studies of nutritional counseling interventions in patients with heart failure showed that nutritional counseling is effective in improving the prognosis of elderly patients with heart failure, despite differences in nutrient composition and food quality.^60^ There is a lack of high‐quality clinical studies on PN or EN support in patients with cardiac insufficiency and malnutrition. A study on 105 elderly patients with chronic heart failure found that the use of EN in conventional treatment not only improved nutritional status and heart function, but also improved immune function, which in turn reduced inflammatory factor levels. The longer the treatment time, the greater the improvement in cardiac function and inflammatory factors.^61^ Use of PN is safe and well tolerated during the perioperative period in patients with heart failure who are treated with ventricular assistance devices.^62^

### 8.2 Chronic obstructive pulmonary disease

Question: How do we provide nutritional support for patients with chronic obstructive pulmonary disease (COPD) in various disease stages?

Recommendation 39: For patients with COPD in the stable stage who have malnutrition, ONSs should be considered. EN formulas with higher fat and protein are recommended, up to 1.5 g/kg/d. It is beneficial to increase the intake of omega‐3 fatty acids and dietary fiber for improving lung function and outcomes. Those patients with a poor appetite can use appetite‐promoting medications to help them consume more nutrition. (Evidence level C, weak recommendation, 93%)

Recommendation 40: For patients with COPD in the acute stage, EN is the first choice for nutritional support, and PN should be administered to those with EN contraindications. When EN cannot provide 60% of the total energy and protein needs of the patient, SPN should be applied. In the PN prescription, fat is recommended to account for 35%‐65% of non‐protein energy and 1.3‐1.5 g/kg/d of amino acids and sufficient micronutrients are recommended. (Evidence level A, strong recommendation, 99%)

Recommendation 41: Nutritional support for mechanically ventilated COPD patients follows the same general principles, but we should avoid overfeeding and control the rate of lipid infusion. (Evidence level C, strong recommendation, 94%)

A study found that the incidence of malnutrition in outpatients with COPD was 25%, compared with over 50% in hospitalized patients and over 60% in patients with acute respiratory failure, of which 43% were patients not on mechanical ventilation, 74% were patients on mechanical ventilation, and 88% of patients on mechanical ventilation for more than 6 days had malnutrition.^63^ COPD patients generally suffer from insufficient nutritional intake, which affects the development and outcome of COPD. A meta‐analysis by Ferreira et al^64^ found that nutritional interventions for COPD patients with low bodyweight significantly improved the patients’ weight, upper arm circumference, and maximum inspiratory expiratory pressure. Given the higher respiratory quotient of carbohydrates, high carbohydrate intake can increase oxygen consumption in patients with COPD, increase patient symptoms, and reduce compliance with nutritional support. A cohort study found that EN with an increased fat‐to‐energy ratio significantly improved nutritional status and respiratory function.^65^ At least 1.5 g/kg/d of protein is recommended to increase muscle mass and promote protein synthesis. A study has found that the prevalence of COPD was lower and lung function was better in a population with high dietary fiber intake.^66^ The use of omega‐3 fatty acids is beneficial for respiratory function and prognoses for the elderly with COPD in the acute stage.

Patients with COPD often suffer from poor appetite due to weak overall status, and patients in the acute stage might have more significant dysphagia due to difficulties with breathing, chewing, and swallowing. One RCT included 128 patients with COPD patients who were underweight (<95% of their ideal weight) and showed that megestrol acetate (800 mg orally once daily) was associated with weight gain and appetite improvement.^67^ There is no evidence of severely impaired bowel function in patients with acute COPD, and EN should be the first choice for these patients. If the EN supply does not reach the target amounts (60%) after 2 days, SPN will be required.^7^ An RCT compared the effect of energy supply by indirect calorimetry and by the use of 25 kcal/kg/d on the length of hospital stay and mortality. It was found that the strict energy supply determined by indirect calorimetry can reduce hospitalization time and hospital mortality by over 50%.^68^ Therefore, it may be more beneficial to measure energy consumption by direct calorimetry and strict planning of energy supply. For most critically ill patients, a safe start for calorie supplementation is 8‐10 kcal/kg/d.^69^ For most stable patients, the target amount of 25‐30 kcal/kg/d should be reached after 1 week. PN could help avoid the risks caused by high‐fat EN, such as gastric emptying and aspiration. Some 35%‐65% of lipids can be used as non‐protein energy sources in the PN prescription. The recommended amino acid target is 1.3‐1.5 g/kg, and vitamins and micronutrients should be simultaneously supplemented.^7^ During the nutrition intervention process, oxygen consumption and carbon dioxide production will increase, which can aggravate the symptoms of dyspnea and increase weaning difficulties in patients with COPD, thus, avoiding overfeeding is important. An RCT on patients with acute respiratory distress syndrome compared a constant infusion of lipids over 6 hours or 24 hours, and the slower infusion rate (24 hours) with a lower shunt fraction was associated with improved arterial oxygen partial pressure (PaO_2_) and fractional inspired oxygen (FiO_2_). Therefore, it was recommended that the fat infusion rate should not exceed 0.05‐1.0 kcal/kg/h.^70^

### 8.3 Alzheimer’s disease

Question: How should we provide nutritional support to patients with Alzheimer’s disease (AD)? Should end‐stage patients with AD be treated with artificial nutritional intervention?

Recommendation 42: ONSs are recommended for patients with AD and malnutrition (Evidence level A, strong recommendation，94%). Short‐term tube‐feeding is recommended only for patients with AD in the event of disease change or emergency. (Evidence level B, weak recommendation, 86%)

Recommendation 43: PN can be given if the feeding tube cannot be tolerated or if EN is contraindicated. Generally, it is not recommended to use artificial nutritional support at the end‐stage of AD. If possible, administration should be determined according to the patient’s wishes. (Evidence level B, weak recommendation, 82%)

Nutritional therapy can improve the nutritional status and general condition of patients with AD^1^, ^7^ and ONSs are beneficial to the nutritional management of these patients as they increase energy and nutrient intake. A study found that patients with AD with a BMI of 25 kg/m^2^ were given ONSs for 3 weeks to 1 year, and the calories were 125‐680 kcal/d. The results suggested that ONSs were well tolerated and helped increase weight and BMI.^71^ Compared to diet counseling and guidance, ONSs are more suitable for elderly patients with early and moderate dementia and can ensure adequate energy and nutrient supply, promote weight gain, and prevent the occurrence and development of malnutrition.^72^

Tube‐feeding in patients with AD is currently controversial. For patients with AD at various stages of the disease, if there are clinical complications (such as pneumonia, stroke, or after surgery), tube‐feeding EN can be applied (assuming it is not contrary to the patient’s wishes) to reduce the nutritional risk of reduced energy intake.^73^ Short‐term tube‐feeding is recommended in patients with AD with fluctuating or emergency conditions, such as pneumonia or airway edema. Patients with AD who have indications for nutritional treatment can use PN if tube‐feeding is not tolerated or EN is contraindicated. The use of PN or EN in the end‐stages of AD is not recommended.^74^

### 8.4 Diabetes mellitus

Question: How do we perform nutritional support treatment for hospitalized elderly patients with diabetes mellitus?

Recommendation 44: The indications for nutritional support for elderly patients with diabetes mellitus are the same as those for patients without diabetes, and EN is the first choice for therapy. Overweight or obese patients do not need to strictly limit energy intake and should maintain weight stability. (Evidence level C, strong recommendation, 94%)

Recommendation 45: Carbohydrate intake should not be excessively restricted in hospitalized elderly patients with diabetes mellitus (Evidence level D, weak recommendation，82%). Choosing carbohydrates with a low glycemic index can also suppress a rapid rise in postprandial blood glucose. (Evidence level C, strong recommendation, 97%)

Recommendation 46: A protein intake of 1.0‐1.5 g/kg/d for elderly patients with diabetes with normal kidney function is recommended. If the patient has renal insufficiency, the protein intake can be reduced to less than 0.8 g/kg/d. (Evidence level C, weak recommendation, 90%)

Recommendation 47: Diabetic EN formulations can be used in elderly patients with diabetes mellitus. (Evidence level A, strong recommendation, 99%)

Recommendation 48: The blood glucose control level of elderly inpatients can be appropriately adjusted to avoid the occurrence of hypoglycemia. At the same time, the risk of acute complications caused by high blood glucose should be considered. (Evidence level A, strong recommendation, 97%)

According to foreign guidelines, it is beneficial for overweight or obese adult patients with type 2 diabetes mellitus to lose a moderate amount of weight through lifestyle changes combined with restriction of energy intake.^7^ However, in view of the prevalence of malnutrition and multiple comorbidities in hospitalized elderly patients, it is more suitable to maintain weight stability without strict restrictions on energy intake for those with diabetes mellitus, even those who are overweight and obese. Any measures to reduce weight can lead to fat‐free weight loss (mainly skeletal muscle weight loss) and can increase the risk of reduced daily activity. The indications for EN and PN for patients with diabetes support are the same as those for patients without diabetes. However, monitoring and treating patients’ blood glucose levels, adjusting patients’ eating habits, and monitoring their metabolic status are important treatment goals.

A systematic review suggested that the protein intake of elderly patients with diabetes mellitus should be 1.0‐1.3 g/kg/d. If combined with renal insufficiency, the protein intake should be reduced to <0.8 g/kg/d. A moderate increase in fat to the energy ratio can help to reduce gluconeogenesis and glycogen consumption and avoid various metabolic complications caused by hyperglycemia.^75^ Excessive restriction of carbohydrate intake can lead to insufficient total energy intake, and bodyweight should be maintained as stably as possible without strict carbohydrate restrictions. Moderate dietary fiber intake can delay the increase in blood glucose and insulin, and improving the blood lipid profile of patients can reduce the risk of cardiovascular disease. When applying standard whole‐protein EN formulas, it is necessary to consider the corresponding insulin therapy and adjust the infusion rate of the nutrient solution.^76^

A systematic review by Elia et al^77^ confirmed that “diabetic‐applicable” EN preparations were beneficial for blood glucose control. Blood glucose levels after feeding were reduced by an average of 1.03 mmol/L compared with patients using standard whole‐protein EN formulas, and the area under the blood glucose curve was also significantly reduced. The study conducted by Mesa García et al^78^ focused on the special EN formulas for diabetes (free of fructose and rich in starch and monounsaturated fatty acids) for elderly patients with diabetes mellitus. They demonstrated that diabetic EN preparations could improve blood glucose control and reduce cardiovascular risk, which is conducive to preventing diabetes‐related complications. Sanz‐Paris et al^79^ used high‐energy EN formulas for patients with diabetes for 1 year of nutritional support for undernourished elderly patients with diabetes, and they found that the formula increased energy intake and glycemic control and improved nutritional indicators.

### 8.5 Perioperative period in elderly patients

Question: Which elderly patients need perioperative nutritional support?

Recommendation 49: There is no need for preoperative nutritional support for elderly patients with a good nutritional status. Elderly patients with severe malnutrition should be given nutritional support for 10‐14 days before the operation. Immuno‐enhanced EN is beneficial for reducing postoperative complications. (Evidence level A, strong recommendation, 97%)

Recommendation 50: The following elderly patients need nutritional support after surgery: patients who have received nutritional support before surgery due to severe malnutrition; patients with severe malnutrition who did not receive nutritional support before surgery; patients with severe traumatic stress and a suspected postoperative inability to eat for more than 7 days; patients with severe postoperative complications requiring fasting for an extended period of time; and those with significantly increased metabolism. (Evidence level B, strong recommendation, 99%)

For elderly patients with good nutritional status or low nutritional risk, preoperative nutritional support is not beneficial,^80^ and guidelines at home and abroad on this issue are clear and consistent. The ESPEN guidelines recommend preoperative nutritional support for patients with severe malnutrition for 7‐14 days, and it is better to postpone the surgery until after that time. The results of the Canadian Oncology Association showed that the ultimate mortality and overall survival rates of elderly patients with non‐emergency colorectal cancer were not affected even if the operation was delayed for 6 weeks after the diagnosis.^81^

Although patients with severe malnutrition can benefit from preoperative nutritional support, if they need to undergo major surgery, it is difficult to endure long‐term nutritional deficiency. In addition, these patients need nutritional support after surgery. For patients with severe malnutrition who have not received nutritional support before surgery, postoperative nutritional support can effectively reduce the incidence of complications and mortality and shorten the length of stay.^82^ Most patients who have surgery can eat independently within 7 days after surgery (>60% of target energy requirements), and their clinical outcomes are not significantly different from those who receive nutritional support. On the other hand, mortality and length of stay are significantly increased in patients who cannot eat for more than 10 days without nutritional support. Adequate postoperative (>60% energy and protein target requirements) and early postoperative (within 48 hours) nutritional support can significantly reduce postoperative hospital stay and costs.^83^ A retrospective analysis of critical surgical patients showed that the risk of death was significantly higher in patients who received less than 60% of their target energy requirements than in patients who received more than 60%.^84^ A systematic review showed that an immunomodulatory formula containing omega‐3 fatty acids, arginine, and RNA for perioperative nutritional supplementation could reduce the incidence of postoperative complications and shorten the length of stay.^85^

Question: How do we provide nutritional support for elderly patients during the perioperative period?

Recommendation 51: ONSs are the first choice for perioperative nutritional support in the elderly, followed by tube‐feeding EN. When tube‐feeding EN cannot be implemented or EN cannot provide sufficient energy and protein, PN should be supplemented or selected. (Evidence level A, strong recommendation, 92%)

Recommendation 52: ONSs should be started within 24 hours after surgery. If ONSs are not possible, tube‐feeding EN should be given. (Evidence level A, strong recommendation, 97%)

Compared with PN, EN can further improve the postoperative clinical outcome of elderly patients. ONSs are the preferred method. PN should be given to elderly patients who need perioperative nutritional support but for whom EN has failed. When EN cannot provide 60% of the body’s target requirements, it is necessary to administer SPN. With an increase in EN tolerance and a decrease in PN requirements, PN can be stopped when the energy and protein provided by EN is greater than 60%.^7^ Early EN after surgery not only provides nutritional substrates, but it also reduces the body’s high catabolic response and insulin resistance, reduces the release of inflammatory factors, maintains the intestinal mucosal barrier and immune function, and prevents intestinal bacterial translocation. The results of multiple studies show that the incidence of complications, such as anastomotic rupture and aspiration, within 24 hours after surgery is not increased compared with fasting patients, and clinical outcomes improve.

### 8.6 Dysphagia

Question: How should a nutritional support treatment plan be formulated for patients with dysphagia?

Recommendation 53: A nutritional support treatment plan should be developed based on the swallowing function classification and nutritional assessment results. (Evidence level C, weak recommendation, 88%)

Recommendation 54: When patients are at nutritional risk or the dysphagia has reached or exceeded Grade 5, and they still cannot achieve adequate nutritional intake after improving food traits and attempting compensatory methods, tube‐feeding EN is recommended. (Evidence level B, strong recommendation, 96%)

The nutritional treatment plan mainly depends on the current nutritional status and swallowing function of the patient. The severity of the primary disease, cognitive function, and compliance should also be considered. The evaluation of swallowing function can make clear the degree and stage of chewing and swallowing ability, and Caiteng’s seven‐grade dysphagia score method is often employed. In the entire phase of dysphagia treatment, the clinical nutritionist should collaborate with the rehabilitation therapist on formulating a “compensatory method” and a standardized “training diet” to assist rehabilitation. Studies have shown that increasing food viscosity can prolong the time of food entering the throat and help improve the nutritional status of patients with dysphagia.^86^ For short‐term EN support, a nasogastric tube can be used. For long‐term (≥4 weeks) EN, PEG should be used for tube‐feeding.^1^

### 8.7 Pressure ulcers

Question: How do we choose a nutritional support method for elderly patients with pressure ulcers?

Recommendation 55: For elderly patients at high risk of pressure ulcers who are at nutritional risk or who have malnutrition, high‐protein ONSs are preferred. Nutrients rich in arginine, vitamin C, and zinc can promote wound healing. (Evidence level B, strong recommendation, 95%)

For elderly patients with pressure ulcers who are well orally fed, the first recommendation is a diet intervention under the guidance of a professional nutritionist. If the intake is still less than 60% of the target amounts, ONSs can be provided. A systematic analysis showed that high‐protein ONSs can significantly reduce the incidence of pressure ulcers in hospitalized patients and have a beneficial effect on the healing of pressure ulcers.^87^ Studies have confirmed that EN can reduce the incidence of pressure ulcers and reduce the cost of hospitalization for high‐risk patients with pressure ulcers, and the use of EN for patients with pressure ulcers can improve the prognosis and reduce total hospitalization costs.^88^ RCT studies have confirmed that nutrients rich in arginine, vitamin C, and zinc can promote wound healing in patients with pressure ulcers compared with typical EN preparations.^89^

### 8.8 Frailty

Question: How do we carry out nutritional support treatment for patients with senile frailty?

Recommendation 56: Increasing energy and protein intake can help improve the nutritional status of frail elderly patients, but it might not improve functional status and mortality. Nutritional supplements rich in essential amino acids can help improve leg muscles and mobility. (Evidence level B, strong recommendation, 92%)

Recommendation 57: The elderly with frailty should have a combined nutritional and exercise intervention. (Evidence level A, strong recommendation, 95%)

Bauer et al^90^ reported that leucine‐rich nutritional supplementation can improve muscle mass and leg mobility in frail elderly people. Comprehensive interventions, including exercise, nutrition, and cognitive function interventions, can reduce frailty. A recent study conducted by Tieland et al^91^ found that resistance supplementation with protein supplementation in the elderly with frailty could improve physical activity.

### 8.9 Sarcopenia in the elderly

Question: How much protein is needed for patients with sarcopenia?

Recommendation 58: An adequate protein supply and reasonable intake patterns can help slow the development of sarcopenia. The recommended amount of protein for the elderly is 1.2‐1.5 g/kg/d. Leucine can increase the rate of skeletal muscle protein synthesis and reduce anabolic resistance. The proportion of whey protein abundant in leucine should be 60% or more. (Evidence level A, strong recommendation, 95%)

Recommendation 59: ONS is the first choice for elderly patients with sarcopenia who are at nutritional risk or who have malnutrition. Supplementation with vitamin D and omega‐3 fatty acids can improve muscle strength and prevent falls. (Evidence level B, strong recommendation, 92%)

Adequate protein supply and reasonable protein intake can overcome the resistance to muscle protein synthesis in the elderly, effectively maintain muscle mass and function, help slow the development of sarcopenia, and might improve health and clinical outcomes. Prospective studies have confirmed that high‐protein intake can reduce the loss of lean body mass by 40%, and increasing protein intake by 20% will reduce the risk of frailty by about 32% in the elderly.^92^

The synthesis of muscle protein varies with the intake of various types of protein, which mainly depends on the content of essential amino acids and branched chain amino acids in the protein, and on the digestion and utilization of food proteins. Leucine plays an important role in stimulating muscle protein synthesis. Ingestion of proteins with a high proportion of leucine, in conjunction with other nutrients, can reverse the decline in muscle mass and function in the elderly.^93^ Whey protein is rich in leucine, which can be digested and absorbed quickly. It can promote muscle synthesis more than soy protein or casein after rest or exercise. The proportion of whey protein in the nutritional formula for patients with senile sarcoma should be 60% or more.^94^

A meta‐analysis suggests that vitamin D supplementation can improve skeletal muscle strength. Vitamin D supplementation has a more pronounced effect on skeletal muscle strength in elderly patients.^95^ Supplementation with omega‐3 polyunsaturated fatty acids can also increase grip strength and protein synthesis in the elderly.^96^ A multicenter RCT study found that ONSs (330 kcal, 20 g protein, 499 IU vitamin D3, 1.5 g HMB, twice per day) for 24 weeks could improve the skeletal muscle strength and muscle mass of malnourished elderly patients with sarcopenia.^33^

### 8.10 End‐stage elderly patients

Question: What are the goals and methods of nutritional management for end‐stage patients?

Recommendation 60: For end‐stage elderly patients, the purpose of intervention is comfort rather than life prolongation, and nutritional assessment and intervention are not recommended. We can support the patient’s desire to drink and eat but should not insist. Gentle care should be given for end‐patients to relieve pain. (Evidence level D, weak recommendation, 86%)

A lack of appetite or an inability to eat in end‐stage elderly patients is a manifestation of the end‐of‐life process, and there is no evidence that artificial nutrition can prolong life or improve the quality of life in these patients. Active nutritional support does not make these patients better, and it might increase the incidence of some side‐effects, such as aspiration, infection, and fluid overload, and it increases medical costs. Moderate eating can make patients comfortable and can be psychologically consoling.^97^ For end‐stage patients, gentle care is important. We should support patients who want to drink and eat, and respect patients’ rights to select whether and what to eat on their own. Active nutritional intervention is not recommended. Soft and easily digestible food can be provided according to the patient’s preferences, and the intake of salt, sugar, and fat should not be restricted too much.

## CONFLICTS OF INTEREST

There are no conflicts of interest to be reported by the authors of this study.
